# Complete Genome Sequence of Mycoplasma bovis Strain XBY01, Isolated from Henan Province, China

**DOI:** 10.1128/MRA.00001-20

**Published:** 2020-04-09

**Authors:** Qingchun Shen, Shijing Sun, Guanlong Xu, Xuezheng Fan, Hui Jiang, Yu Feng, Xiaowei Peng, Liangquan Zhu, Yuming Qin, Jiabo Ding

**Affiliations:** a National/OIE Reference Laboratory for Brucellosis, China Institute of Veterinary Drug Control, Beijing, China; University of Arizona

## Abstract

We report the complete genome sequence of Mycoplasma bovis strain XBY01, which was isolated from a severely diseased young calf in Henan Province, China, in 2019. The genome of XBY01 contains a single circular chromosome of 986,067 bp, with a GC content of 29.30%.

## ANNOUNCEMENT

Mycoplasma bovis is a wall-less bacterium with zoonotic potential, belonging to the family *Mycoplasmataceae* in the class *Mollicutes*. M. bovis is an important pathogen that causes pneumonia and otitis media in young calves, and infection results in mastitis and arthritis in cattle, leading to substantial economic losses to the cattle industry worldwide (1, 2). Virulence-associated factors of M. bovis, including lipoproteins and secreted proteins, might contribute to bacterial adhesion and invasion into host cells, as well as subsequent survival and dissemination within hosts (3).

In this study, we obtained the complete genome sequence of M. bovis strain XBY01, which caused respiratory diseases in cattle on a farm in Henan Province, China, in 2019. M. bovis strain XBY01 was isolated from the lungs of a young calf that had died from serious pneumonia. To isolate M. bovis, small pieces of the affected parts of the lungs were homogenized in 10 ml of *Mycoplasma* broth (BD, Difco) and cultured at 37°C. On the third day of incubation, when a slight color change of the broth occurred, the cultures were inoculated onto pleuropneumonia-like organism (PPLO) agar (BD Difco); the cultures were incubated at 37°C in 5% CO_2_ for 3 days until visible colonies appeared (4). The cultures were purified twice by single-colony isolation and identified with PCR targeting the *uvrC* gene of M. bovis (5). The purified isolate was named M. bovis strain XBY01 and was cultured in PPLO broth and stored at −80°C after the addition of 3% glycerol.

The fresh PPLO broth culture of M. bovis strain XBY01 was centrifuged at 12,000 rpm for 10 min, and the cell pellets were used to isolate genomic DNA with a bacterial DNA kit (Omega Biotech, Beijing, China) according to the manufacturer’s instructions. The genomic DNA was quantified by using a TBS-380 fluorometer (Turner BioSystems, Inc., Sunnyvale, CA). A DNA sample with high quality (optical density at 260 nm/optical density at 280 nm, 1.8 to 2.0; >6 μg) was used to construct a fragment library.

We used various software packages for our analyses, as described below. Default parameters were used for all software unless otherwise specified. We used an Illumina TruSeq Nano DNA kit to construct a sequencing library. The parameters for the Illumina data analysis software were as follows: the read number was 15,425,194, the read length was 150 bp × 2, the sequence reads totaled 2,313,779,100 bp, and the coverage was 2,346×. Sequencing was performed on the Illumina HiSeq platform in paired-end mode (300 cycles). For Illumina paired-end sequencing of the genome, paired-end libraries with an insert size of ∼400 bp were prepared and qualified following Illumina’s standard genomic DNA library preparation procedures. For Pacific Biosciences sequencing, whole-genome shotgun libraries with inserts of 20 kb were generated and sequenced on a Pacific Biosciences RS instrument, using standard methods. The software parameters we used for Pacific Biosciences sequencing were as follows: the read number was 86,619, the read *N*_50_ value was 6,272 bp, the sequence reads totaled 376,136,235 bp, and the coverage was 381×. An aliquot of 8 μg DNA was centrifuged in a g-TUBE (Covaris, MA) at 6,000 rpm for 1 min. DNA fragments were then purified, end repaired, and ligated with SMRTbell sequencing adapters following the manufacturer’s recommendations (Pacific Biosciences). The resultant sequencing libraries were purified three times using 0.45 volumes of Agencourt AMPure XP beads (Beckman Coulter Genomics, MA) following the manufacturer’s recommendations.

The XBY01 genome was sequenced using a combination of Illumina and PacBio RS sequencing platforms. The Illumina data were used to evaluate the complexity of the genome and to correct the PacBio long reads. First, we used ABySS to perform genome assembly with multiple *k*-mer parameters, and we accepted the assembly with the longest *N*_50_ value, which was considered the optimal result (6, 7). GC content and genome size information were calculated by custom Perl scripts, which allowed us to judge whether the DNA sample was contaminated. Second, Canu v1.8 (https://github.com/marbl/canu) was used to assemble the corrected PacBio long reads and next-generation sequencing data (8). Finally, GapCloser software (https://sourceforge.net/projects/soapdenovo2/files/GapCloser) was used to fill the remaining local inner gaps and to correct the single-base polymorphisms for the final assembly results.

The complete genome sequence of M. bovis strain XBY01 was obtained, with a mean GC content of 29.3%. The NCBI PGAP was used to annotate this genome (9). A total of 1,867 gene sequences were present in XBY01, including two sets of 16S rRNAs and 34 tRNAs, and the total gene length was 709,566 bp, with a mean GC content of 29.8%.

The genome size of XBY01 was 986,067 bp, which was comparable to the values for strain 08M (GenBank accession no. CP019639) (1,016,753 bp) (10), strain PG45 (NC_014760.1) (1,003,453 bp) (11), strain HB0801 (NC_018077.1) (991,653 bp) (12), and strain Hubei-1 (NC_015725.1) (948,153 bp) (13).

### Data availability.

The genome sequence and associated data for M. bovis strain XBY01 were deposited under GenBank accession no. CP045797, BioProject accession no. PRJNA587561, SRA accession no. SRX7531776 and SRX7531777, and BioSample accession no. SAMN13193745.
